# Comparative Study on the Sperm Proteomes of Horses and Donkeys

**DOI:** 10.3390/ani14152237

**Published:** 2024-07-31

**Authors:** Hong Ren, Xin Wen, Qianqian He, Minna Yi, Manglai Dugarjaviin, Gerelchimeg Bou

**Affiliations:** 1 Inner Mongolia Agricultural University, Hohhot 010018, China; renhong1980@126.com (H.R.); wenxin618@imau.edu.cn (X.W.); qianqianhe202309@163.com (Q.H.); yiminna2020@163.com (M.Y.); 2 Inner Mongolia Key Laboratory of Equine Science Research and Technology Innovation, Inner Mongolia Agricultural University, Hohhot 010018, China

**Keywords:** comparative, sperm, proteome, viability, horse, donkey

## Abstract

**Simple Summary:**

This study identified the protein expression profiles of horse and donkey sperm and investigated the effect of sperm proteins on sperm viability. In short, we first assessed the viability of horse and donkey sperm, revealing higher viability in donkey sperm compared to horse sperm. Subsequently, 4D-DIA protein-sequencing technology was employed to identify 3436 proteins expressed in horse sperm and 3404 proteins expressed in donkey sperm, with 73 proteins specific to horse sperm, and 41 specific to donkey sperm. Further analysis established a correlation between sperm proteins and sperm viability. These findings are significant in elucidating the reproductive variances and evolutionary relationships between horses and donkeys.

**Abstract:**

The reproductive performance of horse sperm and donkey sperm has been reported to differ. Sperm proteins play a crucial role in sperm viability and fertility. Although differences between species are known, no prior study has investigated disparities in the sperm proteome between horses and donkeys. Therefore, this study characterized and compared the sperm proteomes of horses and donkeys using 4D-DIA mass spectrometry technology. We identified 3436 proteins in horse sperm and 3404 proteins in donkey sperm. Of these, 3363 proteins were expressed in both horse and donkey sperm, with 73 proteins being specifically expressed in horse sperm, and 41 in donkey sperm. According to data analysis, donkeys exhibited a greater percentage of motility and progressive movement in straight-line sperm than horses, as well as lower percentages of static and slow sperm than horses. Joint analysis of the results from the horse and donkey sperm proteomes and their CEROS II-read parameters demonstrated a possible association between sperm proteins and their sperm viability patterns. These findings suggest that there are discrepancies in the expression levels and protein compositions of horse and donkey sperm and that certain specific proteins may be responsible for the differences in performance between these two species.

## 1. Introduction

Horses and donkeys are significant livestock species and also serve as crucial genetic resources with unique value in biological research [1,2]. While both horses and donkeys belong to the equine family, there are notable genetic differences between them [3,4,5].

The vitality of male reproductive cells directly impacts the reproductive capacity of animals [6,7,8,9]. Sperm proteins are integral components of sperm cells and are involved in the formation, maturation, morphology, motility, and fertilization capacity of sperm in humans [10,11,12]. Wu et al. indicated that the proteins AKAP4, ODF1, ODF2, GAPDHS, SPESP1, and ACTRT2 play an important role in sperm concentration and motility [13]. Fang and Blommaert et al. substantiated Akap4’s indispensable roles in the structure, chemotaxis, and gradual maturation of sperm flagella [14,15]. CABYR is essential for fibrous sheath integrity and progressive motility in mouse and human spermatozoa [16,17]. ODF1 is the main protein in the tail auxiliary fibers of mammalian sperm, which can connect the head and tail of sperm and are crucial to the gradual movement of sperm to reach the oocyte for fertilization [18]. Therefore, sperm proteins play a crucial role in determining sperm function [19].

Proteomic technology enables a more comprehensive and in-depth study of the molecular diversity of sperm proteins [20,21,22]. Four-dimensional data-independent acquisition (4D-DIA) proteomics technology uses parallel accumulation–serial fragmentation (PASEF) combined with the DIA acquisition mode of a timsTOF Pro2 series mass spectrometer to conduct differential quantitative proteomic analysis [23,24]. This mode offers the advantage of acquiring DIA data without compromising the window cycling speed. This simultaneously reduces spectral complexity and improves ion utilization, resulting in a comprehensive enhancement of proteomics in terms of coverage depth and sensitivity [25,26]. This method can be used to evaluate the clinical response of iricoxib in the early treatment of osteoarthritis and predict the efficacy of glucocorticoids in primary immune thrombocytopenia in children [27,28]. In the field of reproductive development, the 4D-DIA method has been applied to detect frozen–thawed spermatozoa with different levels of freezability in dairy goats and to analyze the proteomic landscape of human spermatozoa [29,30,31].

Previous research has demonstrated the utility of proteomics in assessing the function of different animal sperm [32,33,34]. James and Giorgia et al. conducted proteomics on seminal plasma from donkeys and horses to analyze the relationship between seminal plasma components and sperm motility. These findings suggested that seminal plasma proteins may be associated with sperm viability [35,36,37]. There should be a more pronounced correlation between sperm protein and sperm vitality [38,39,40]. However, there are currently almost no comparative studies of the sperm protein proteomes of horses and donkeys to determine the correlation between sperm proteins and sperm viability in these two species.

In this study, we employed the 4D-DIA proteomics method to analyze the protein profiles of horse and donkey sperm, identifying both differences and similarities in their protein composition, as well as their correlation with sperm viability. This investigation offers valuable insights into the molecular diversity of horse and donkey sperm proteins, as well as the correlation between sperm proteins and sperm vitality, and contributes to the development of equine breeding research.

## 2. Materials and Methods

### 2.1. Experimental Design

**Figure 1:**
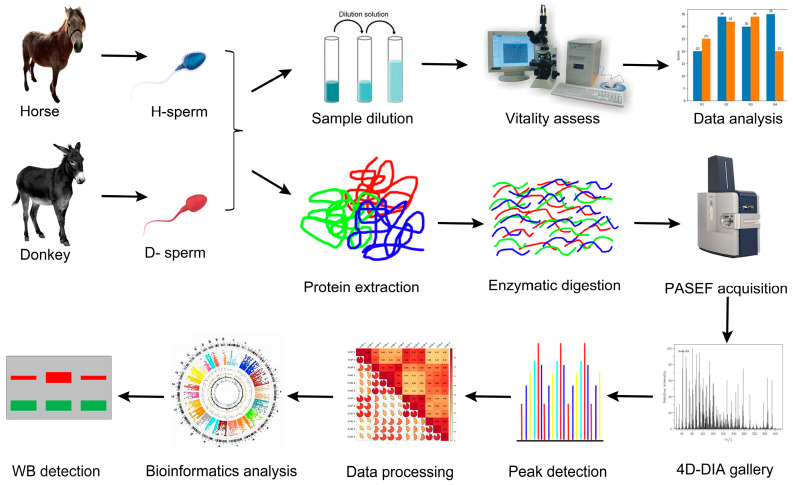
Schematic of the experimental design and workflow. The sperm vitality of the horses and donkeys was assessed, and quantitative proteomic analysis was conducted on sperm proteins using a 4D DIA-based LC–MS/MS method. Following extensive statistical analysis of the proteomic data, a few key proteins were chosen for validation through Western blotting.

Sperm samples from horses and donkeys were collected separately. A portion of the samples was diluted for sperm viability testing, while another portion underwent protein extraction, enzymatic digestion, PASEF collection, a 4D-DIA library search, peak detection, data processing, and bioinformatics analysis. Subsequently, Western blotting experiments were conducted to validate the expression of key proteins in horse and donkey sperm. The experimental design and workflow are illustrated in Figure 1.

### 2.2. Animals and Chemicals

The research was conducted at the Sports Horse Training Center of Inner Mongolia Agricultural University during the breeding season from June to August 2023. The Mongolian stallions had an average body weight of 300 kg (range: 290–310 kg) and an average age of 5 years (range: 4–6 years), while the North China donkeys had an average body weight of 200 kg (range: 190–210 kg) and an average age of 5 years (range: 4–6 years). The animals were individually housed in comfortable boxes with straw bedding, received forage twice a day at approximately 2% of their body weight, and consumed concentrates daily at a rate of 3 ± 1 kg/day; water was available ad libitum. All research procedures complied with regulations and were approved by the Animal Care Commission of Inner Mongolian Agriculture University. The chemicals used in this study were all obtained from Sigma–Aldrich (St. Louis, MO, USA) unless otherwise stated.

### 2.3. Sperm Sources and Preparation

Sperm were obtained from three Mongolian stallions and three North China donkeys, all of which had been proven to be fertile based on the successful siring of multiple foals in the past two years. Sperm were collected using an artificial vagina with an inner lining and an in-line filter on a prosthesis. The sperm concentration was determined using the NucleoCounter^®^ SP-100™ (ChemoMetec, Bohemia, New York, NY, USA). The sperm were treated according to the method described by Xu [41]. Briefly, the initial sperm motility after collection was maintained at 80% or above, with a minimum percentage of normal morphology sperm at 80% and a sperm density exceeding 3 × 10^7^/mL. The sperm were separated by centrifugation at 1000× *g* for 20 min at 4 °C. Sperm were washed twice using PBS at 1000× *g* for 20 min at 4 °C. Subsequently, the sperm were resuspended in PBS and then divided into microcentrifuge tubes, which were centrifuged at 16,000× *g* for 10 min at 4 °C to completely remove the supernatant. The resulting sperm pellet was rapidly frozen in liquid nitrogen and stored at −80 °C for later use.

### 2.4. Sperm Morphology and Viability Assessment

Sperm viability was evaluated using a CEROS II analyzer (Hamilton Thorne, Beverly, MA, USA), which provided kinematic parameters such as both sample means and individual sperm tracks. Briefly, 5 μL of each horse and donkey sperm sample (diluted in PBS at a concentration of 5 × 10^7^ spermatozoa/mL) was loaded onto a Makler chamber (Sefi Medical Instruments, Haifa, Israel). Subsequently, the samples were assessed under a 40× phase contrast objective using an Axiolab A1 microscope (Olympus, Tokyo, Japan) with a minimum count of 1000 sperm cells per analysis. The parameters to be considered included the percentages of static, progressive, motile, and slow sperm. A phase contrast microscope was also used to observe horse and donkey sperm morphology at a 40× magnification. Morphological characteristics included bent tail, coiled tail, DMR, distal droplet, and proximal droplet.

### 2.5. Protein Extraction and Digestion

Following sperm morphology and vitality data, we performed proteomic assessment on the sperm. An amount of 1 mM phenylmethanesulfonylfluoride or phenylmethylsulfonyl fluoride (PMSF), a protease inhibitor, was added to the collected horse and donkey sperm samples, followed by centrifugation at 4500× *g* for 10 min to collect the supernatant. The protein concentration was determined using a BCA assay kit. Subsequently, the supernatant was transferred to a 10 Kd ultrafiltration tube, and the ultrafiltration process was repeated to concentrate the protein solution within the tube. Based on the protein concentration, an equal volume of protein solution was taken and adjusted to 200 µL with 8 M urea. Subsequently, the protein was subjected to reduction using 10 mM DTT at 37 °C for 45 min in a dark environment, followed by alkylation with 50 mM IAM for 15 min. The addition of four times the volume of precooled acetone led to protein precipitation at −20 °C for 2 h. After centrifugation, the dried protein precipitate was reconstituted in a solution containing 200 µL of 25 mM ammonium carbonate and 3 µL of trypsin (Promega, Madison, WI, USA) and then digested overnight at 37 °C. After digestion, peptide desalting was performed using a C18 column, followed by vacuum concentration and reconstitution in a solution containing 0.1% formic acid.

### 2.6. LC–MS/MS Detection

The sample was separated using a NanoElute UHPLC system (Bruker Daltonic, Billerica, MA, USA) at a nanoliter flow rate. Phase A consisted of a 0.1% formic acid aqueous solution, while phase B consisted of a 0.1% formic acid acetonitrile solution. The sample was automatically loaded onto an analytical column (IonOpticks, Fitzroy, VIC, Australia). The temperature of the analytical column was maintained at 50 °C using an integrated column oven. An injection volume of 200 ng and a flow rate of 300 nL/min were utilized with a gradient duration of 40 min. After the sample mixture was chromatographically separated, mass spectrometry data were collected using the ddaPASEF mode of the timsTOF Pro2 mass spectrometer to establish appropriate acquisition windows for the diaPASEF method. The effective gradient duration was 40 min, with positive ion detection and a parent ion scan range of 100–1700 *m*/*z*. The ion mobility drift range (1/k0) was 0.85–1.3 Vs/cm^2^, and the ion accumulation and release time was set at 100 ms, achieving an almost 100% ion utilization rate.

### 2.7. Mass Spectrometry Data Analysis

The raw data collected by mass spectrometry were preprocessed, using specific algorithms to identify peaks in the mass spectrometry data. Subsequently, the library-free DIA-NN (v1.8.1) search library software was used to determine the substances corresponding to the identified peaks. Following peak matching, a spectrum library was generated based on deep learning parameters, and this spectrum library was employed for reanalyzing the DIA data to achieve protein quantification, precursor ions, and protein-level FDR filtering at 1%. Subsequently, differentially expressed proteins (DEPs) were selected using Student’s *t* test, with proteins exhibiting *p* values < 0.05 and fold changes of  >1.2 being selected.

### 2.8. Bioinformatics Analysis

The molecular functions (MFs), biological processes (BPs), and cellular components (CCs) of the DEPs were annotated using Blast2GO software (V6.0). Additionally, the DEPs were annotated utilizing the Kyoto Encyclopedia of Genes and Genomes (KEGG) pathway database. Subsequently, the Interpro database was used for the functional domain annotation analysis of the DEPs. Fisher’s exact test was utilized to identify significantly enriched Gene Ontology (GO) terms, KEGG pathways, and functional domains.

### 2.9. PPI and Correlation Matrix Construction

The protein–protein interaction (PPI) network was constructed by the Search Tool for the Retrieval of Interacting Genes (STRING) database and visualized with Cytoscape software (version 3.7.2). The correlation matrix was generated by calculating Spearman’s correlation coefficients between the sperm proteins identified in donkeys and horses and their respective vitality parameters.

### 2.10. Western Blot

Horse and donkey sperm were lysed in 2× loading buffer (Thermo Fisher Scientific, Waltham, MA, USA), the proteins were separated on a 12% polyacrylamide gel, and then the proteins were transferred to a nitrocellulose membrane. The membrane was blocked in nonfat milk powder for 1 h at 37 °C and then incubated with antibodies against AKAP4 (1:500; 24986-1-AP, Proteintech, Wuhan, China), CABYR (1:500; 12351-1-AP, Proteintech), ACADSB (1:500; 13122-1-AP, Proteintech), S100A12 (1:500; 16630-1-AP, Proteintech), and tubulin (1:1000; ab6046, Abcam, Cambridge, MA, USA) overnight at 4 °C. After washing with TBST, the membrane was incubated with a secondary antibody at room temperature for 1.5 h. The protein bands were visualized with enhanced chemiluminescence (ECL) Plus (GEL, San Francisco, CA, USA) and visualized with a Gel Doc XR+ Gel Documentation System (Bio-Rad, Hercules, CA, USA). The unedited and uncropped original Western blotting image is shown in Figure S1, and the ratio of target protein to internal reference is shown in Table S1.

### 2.11. Statistical Analysis

At least three independent experiments were performed, and GraphPad Prism 9.0 software (GraphPad Prism, La Jolla, CA, USA) was used to perform one-way ANOVA and Student’s *t* test. The results are shown as the means ± standard deviations (SDs). The specific *p* values for the other data are provided in the figures.

## 3. Results

### 3.1. Evaluation of Horse and Donkey Sperm Morphology and Vitality

**Figure 2:**
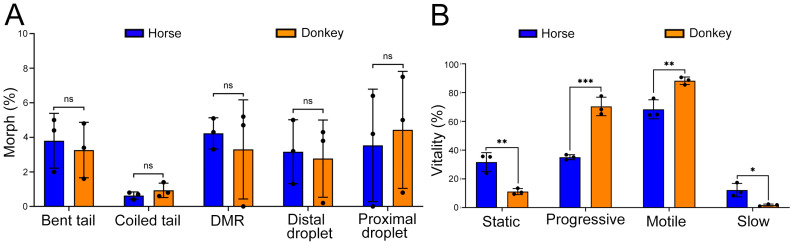
Evaluation of horse and donkey sperm morphology and vitality. (**A**) CASA features of morphological parameters in horse and donkey sperm. (**B**) CASA features of vitality parameters in horse and donkey sperm. No significant difference is indicated by ns (*p* > 0.05), a single asterisk (*) indicates a statistical difference at *p* < 0.05, a double asterisk (**) indicates a significant statistical difference at *p* < 0.01, and three asterisks (***) indicates an extremely significant statistical difference at *p* < 0.001.

In this study, we collected sperm from horses and donkeys separately, assessed sperm morphology and vitality using the CASA system, and conducted quantitative proteomic analysis of sperm proteins employing a 4D-DIA based LC-MS/MS method. Following a comprehensive assessment of the morphological and vital characteristics of horse and donkey sperm, it was determined that there are no significant differences in morphological parameters (bent tail, coiled tail, DMR, distal droplet, or proximal droplet) between horse and donkey sperm (*p* > 0.05) (Figure 2A). However, notable differences were observed in the vitality of the spermatozoa of these two species. Specifically, the percentages of progressive and motile parameters of donkey sperm were significantly greater than those of horse sperm; conversely, the percentages of static and slow parameters were notably lower (*p* < 0.05) (Figure 2B). This indicates a significant disparity in sperm viability between horse and donkey sperm.

### 3.2. Displaying the Horse and Donkey Sperm Proteome

**Figure 3:**
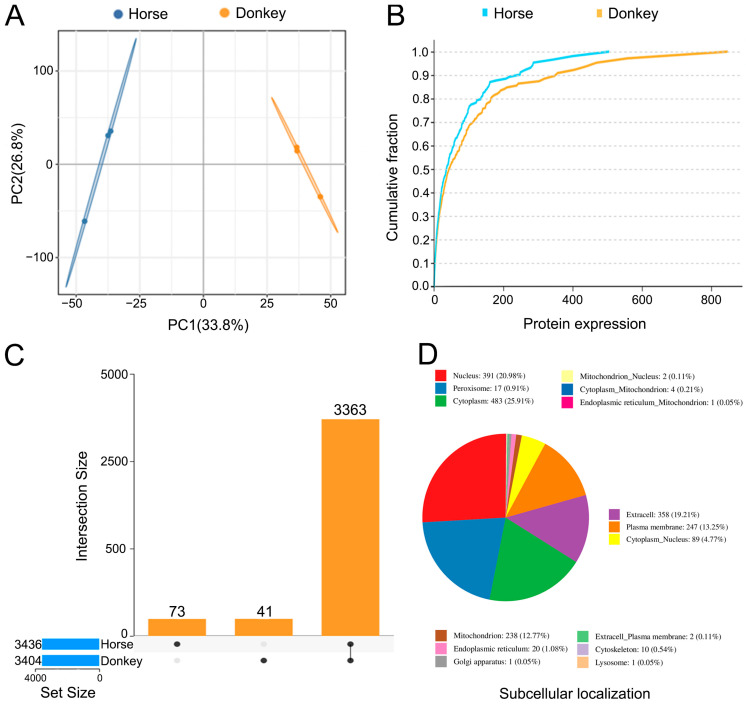
Display of the horse and donkey sperm proteomes. (**A**) PCA of the horse and donkey sperm proteomes. (**B**) CDC of the horse and donkey sperm proteomes. (**C**) UpSet plot of the horse and donkey sperm proteomes. (**D**) Subcellular localization of the horse and donkey sperm proteomes.

In this study, 4D-DIA quantitative proteomics technology was used to compare the protein composition and expression in horse and donkey sperm. The sperm protein samples from each species were subjected to three independent biological replicates. Principal component analysis (PCA) revealed distinct distribution patterns between the protein samples from horse and donkey sperm, suggesting differences in their protein composition or expression profiles (Figure 3A). The cumulative distribution curve (CDC) illustrates the frequency disparities between the horse sperm and donkey sperm datasets across various data intervals. The blue line is positioned above the 0–550 range, indicating a generally higher protein expression in the horse sperm group within this range, with a maximum protein expression of 550. Within the 550–830 range, there was a rapid increase in protein expression in the donkey sperm group, suggesting relatively higher protein expression levels at this stage and a maximum protein expression of 830 (Figure 3B). The UpSet diagram illustrates that the horse sperm sample contains 73 unique proteins, whereas the donkey sperm sample contains 41 unique proteins. Additionally, 3363 proteins were shared between horse and donkey sperm (Figure 3C). Among these proteins, 25.91% are predominantly localized in the cytoplasm and 20.98% are located within the cell nucleus; together, these two categories account for nearly half of the total protein count. In addition, some proteins localize to the plasma membrane, extracellular space, and other cellular compartments (Figure 3D). This indicates distinct sperm protein profiles between horses and donkeys.

### 3.3. Presentation of Horse and Donkey Sperm CEPs

**Figure 4:**
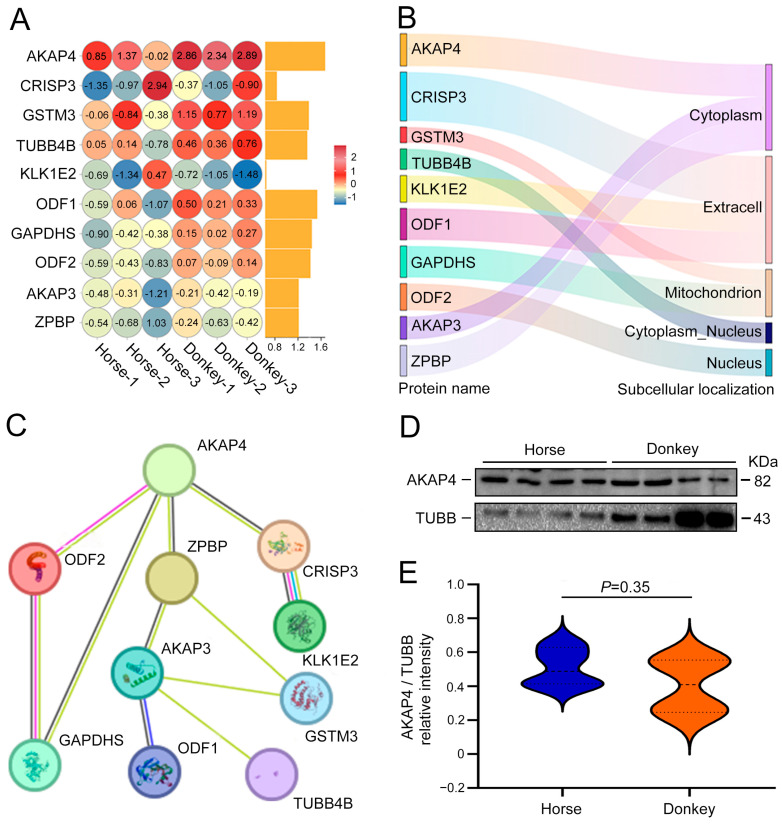
Presentation of horse and donkey sperm CEPs. (**A**) The expression levels of the top 10 CEPs in horse and donkey sperm. (**B**) Subcellular localization of the top 10 CEPs in horse and donkey sperm. (**C**) The PPIs of the top 10 CEPs in horse and donkey sperm. (**D**) AKAP4 protein expression in horse and donkey sperm. (**E**) Quantification of the relative intensity of AKAP4/TUBB. TUBB protein expression was used as an internal control. The data are expressed as the means ± SDs. *p* < 0.05 was considered to indicate a statistically significant difference.

To characterize the properties of horse and donkey sperm proteins, we conducted a comprehensive analysis of the sperm proteome sequencing data from these two species, identifying those that are co-expressed proteins (CEPs) in both horse and donkey sperm. We found that the top 10 proteins with the highest expression levels were the same in both horse and donkey sperm, namely, proteins AKAP4, CRISP3, GSTM3, TUBB4B, KLKIE2, ODF1, GAPDHS, ODF2, AKAP3, and ZPBP. Furthermore, the expression levels of the donkey sperm proteins in this top 10 list were notably greater than those of the horse sperm proteins, with the exception of the CRISP3 and KLK1E2 proteins (Figure 4A). Among them, AKAP4, AKAP3, and ZPBP are localized in the cytoplasm, while CRISP3, KLK1E2, and ODF1 are extracellular. GAPDHS and GSTM3 predominantly reside in the mitochondria, ODF2 is located in the nucleus, and TUBB4B is localized in both the nucleus and cytoplasm (Figure 4B). We observed interactions among all of the top 10 proteins, with AKAP4 showing connections with the majority of the other proteins (Figure 4C). This finding suggested that AKAP4 may be a pivotal protein. Furthermore, considering previous reports linking AKAP4 protein to sperm viability and fertility in other species, we employed Western blotting to assess the expression of AKAP4 protein in horse and donkey sperm. AKAP4 protein was highly expressed in both horse and donkey sperm, but the difference was not significant (*p* > 0.05) (Figure 4D,E). The aforementioned evidence indicates that, while the majority of proteins in horse and donkey sperm are shared, there may be differences in their expression trends, potentially accounting for the difference in sperm viability between these two species.

### 3.4. Classification of Horse and Donkey Sperm SEPs

**Figure 5:**
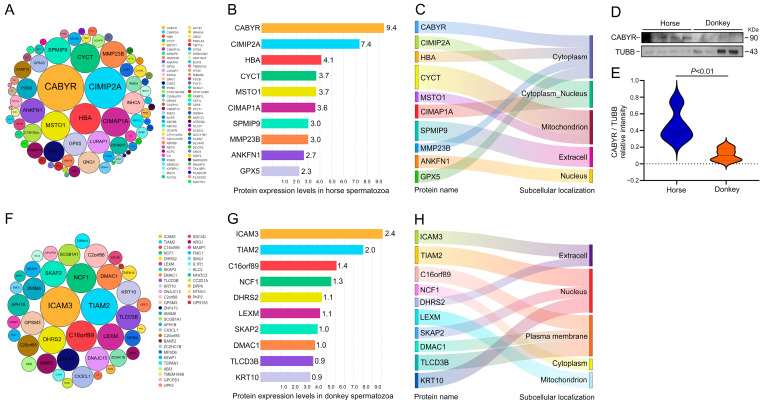
Classification of horse and donkey sperm SEPs. (**A**) A total of 73 SEPs in horse sperm, with the different-sized circles representing the expression levels of proteins. (**B**) The expression levels of the top 10 SEPs in horse sperm. (**C**) Subcellular localization of the top 10 SEPs in horse sperm. (**D**) CABYR protein expression in horse sperm. (**E**) The quantified relative intensity of CABYR/TUBB. (**F**) A total of 41 SEPs in donkey sperm, with the different-sized circles representing the expression levels of proteins. (**G**) The expression levels of the top 10 SEPs in donkey sperm. (**H**) Subcellular localization of the top 10 SEPs in donkey sperm. TUBB protein expression was used as an internal control. The data are expressed as the means ± SDs. *p* < 0.05 was considered to indicate a statistically significant difference.

After confirming the presence of CEPs in horse and donkey sperm, we aimed to investigate the specific-expressed proteins (SEPs) in both species. The results revealed a total of 73 proteins that were specifically expressed in horse sperm (Figure 5A). The top 10 proteins identified were CABYR, CIMIP2A, HBA, CYCT, MSTO1, CIMAP1A, SPMIP9, MMP23B, ANKFN1, and GPX5 (Figure 5B). Among these proteins, CABYR, HBA, MMP23B, and GPX5A are predominantly located in the cytoplasm; CYCT and MSTO1 are localized in the mitochondria; SPMIP9 is distributed extracellularly; ANKFN1 is found within the nucleus; and CIMIP1A and CIMIP2A are present in both the cytoplasm and nucleus (Figure 5C). The CABYR protein was validated by proteome sequencing and analysis accuracy. CABYR exhibited high expression in horse sperm, while its expression was minimal in donkey sperm (*p* < 0.05) (Figure 5D,E). Subsequently, the analysis revealed the presence of 41 CEPs in donkey sperm (Figure 5F). Among these, ICAM3, TIAM2, C16orf89, NCF1, DHRS2, LEXM, SKAP2, DMAC1, TLCD3B, and KRT10 were identified as the top 10 (Figure 5G). Among these proteins, ICAM3 and DHRS2 are predominantly located in the extracellular space; TIAM2, SKAP2, and KRT10 are primarily localized in the nucleus; DMAC1 and TLCD3B are located in the plasma membrane; NCF1 is found within the nucleus; and LEXM is located in the mitochondria (Figure 5H). Considering the extracellular localization of the ICAM3 protein, we planned to study the protein expression of TIAM2. However, due to the low expression level of TIAM2 protein in donkey sperm, no TIAM2 bands were detected in Western blotting experiments. Nonetheless, these findings also indicates the presence of distinct protein expression profiles in horse and donkey sperm, which may represent an additional potential factor contributing to the disparity in sperm viability between horses and donkeys.

### 3.5. Comparison of DEPs between Horse and Donkey Sperm

**Figure 6:**
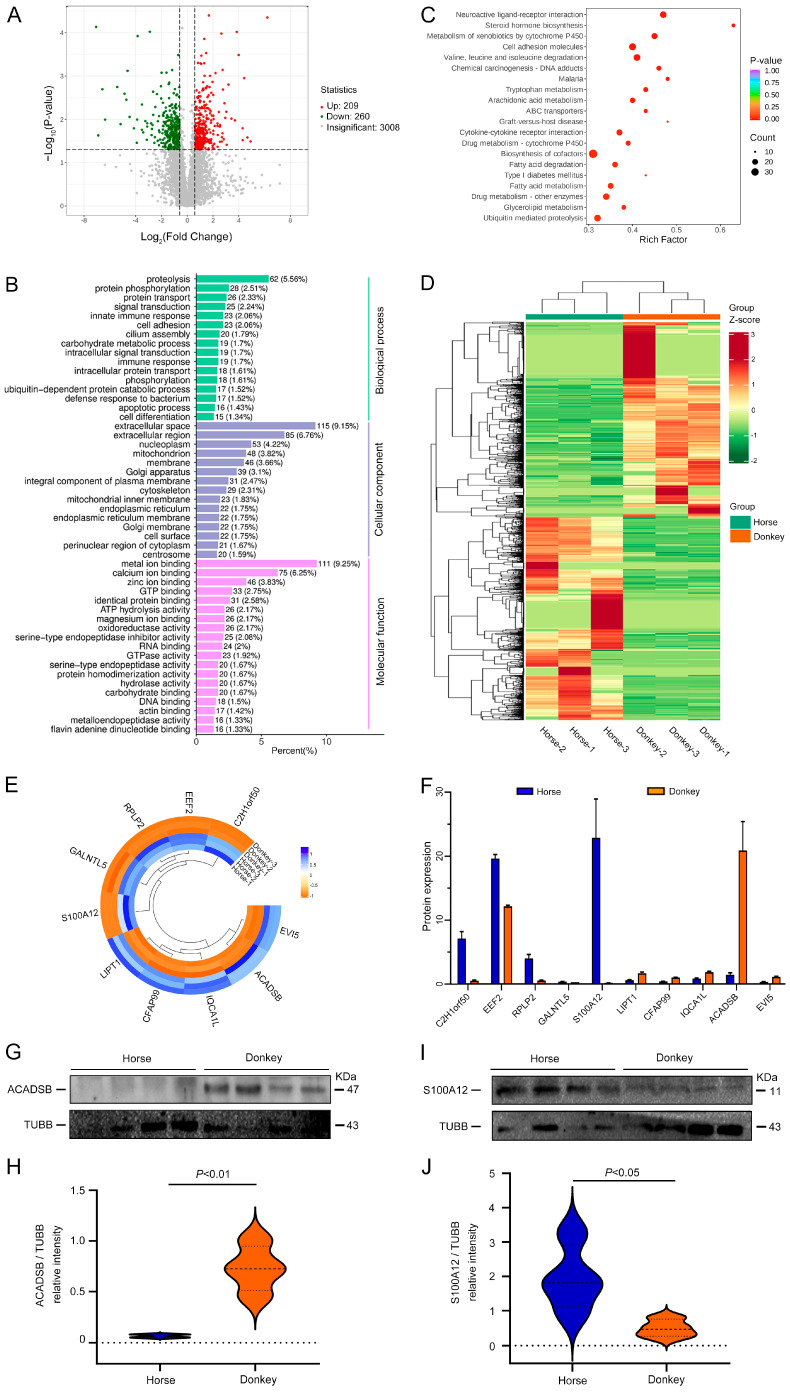
Comparison of DEPs between horse and donkey sperm. (**A**) Volcano plot of horse and donkey sperm DEPs, with upregulated and downregulated proteins represented by green and red dots, respectively. (**B**) GO enrichment analysis of the DEPs in horse and donkey sperm. (**C**) KEGG pathways of the DEPs in horse and donkey sperm. (**D**) Heatmap of all DEPs involved in the top 10 signaling pathways in horse and donkey sperm. (**E**) Circle diagram of the top 10 DEPs in horse and donkey sperm. (**F**) The expression of the top 10 DEPs in horse and donkey sperm. (**G**) ACADSB protein expression in horse and donkey sperm. (**H**) The quantified relative intensity of ACADSB/TuBB. (**I**) S100A12 protein expression in horse and donkey sperm. (**J**) Quantification of the relative intensity of S100A12/TuBB. TUBB protein expression was used as an internal control. The data are expressed as the means ± SDs. *p* < 0.05 was considered to indicate a statistically significant difference.

Differential analysis of the proteomic data from horse and donkey sperm revealed 3477 DEPs. Among them, 209 upregulated proteins are represented in red, 260 downregulated proteins are represented in green, and 3008 proteins showed no differential expression and are depicted in gray (Figure 6A). The DEPs between horse and donkey sperm were functionally annotated against the UniProt databases and then grouped based on GO enrichment: BP, CC, and MF. Proteins enriched in BP were involved in protein degradation, phosphorylation, transport, and signal transduction. The majority of proteins within the CC group were enriched in extracellular regions, cytoplasmic membranes, and mitochondria. Most proteins associated with MFs were related to calcium ion binding, zinc ion binding, and GTP binding. These observations suggest the potential involvement of DEPs in diverse processes and binding activities within horse and donkey sperm (Figure 6B). We further analyzed the DEPs using the KEGG database. In total, we identified 29 enriched KEGG pathways across different species. Among the top 10 KEGG pathways were neuroactive ligand–receptor interactions, steroid hormone biosynthesis, the metabolism of xenobiotics by cytochrome P450, cell adhesion molecules, valine, leucine and isoleucine degradation, chemical carcinogenesis–DNA adducts, malaria, tryptophan metabolism, arachidonic acid metabolism, and the ABC transporter signaling pathway (Figure 6C). The top 10 KEGG signaling pathways were associated with a total of 185 DEPs (Figure 6D). Among the top 10 DEPs were C2H1orf50, EEF2, RPLP2, GALNTL5, S100A12, LIPT1, CFAP99, IQCA1L, ACADSB, and EVI5 (Figure 6E). The expression of the first five proteins was significantly greater in horse sperm than in donkey sperm, while the expression of the last five proteins exhibited the opposite pattern (Figure 6F). We selected DEPs with high expression levels from the proteomic analysis of sperm proteins from horses and donkeys and performed immunoblotting experiments. The results demonstrated that the expression of the ACADSB protein in donkey sperm was significantly greater than in horse sperm (Figure 6G,H). However, the expression of S100A12 in horse sperm was significantly greater than in donkey sperm (Figure 6I,J). This finding is consistent with the sequencing results, validating the accuracy of the proteomic and analytical findings.

### 3.6. Correlations between Horse and Donkey Sperm Protein Levels and Sperm Viability

**Figure 7:**
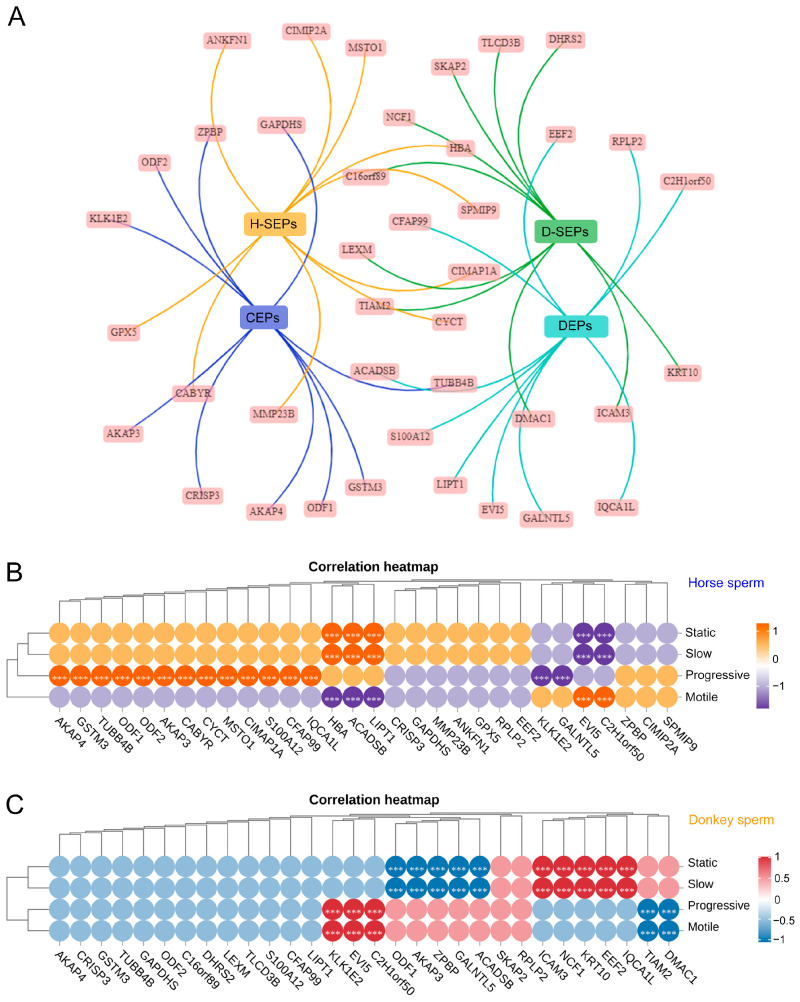
Correlation between horse and donkey sperm proteins and sperm viability. (**A**) The PPI network of horse and donkey sperm CEPs, SEPs, and DEPs. H-SEPs represent the SEPs of horse sperm. D-SEPs represent the SEPs of donkey sperm. (**B**) The correlation between horse sperm protein and sperm viability. The scale (1 to −1) indicates whether the correlation is positive (orange) or negative (purple). (**C**) The correlation between donkey sperm protein levels and sperm viability. The scale (1 to −1) indicates whether the correlation is positive (red) or negative (blue). *** indicates a statistically significant difference.

Based on these findings, we constructed PPI diagrams for the top 10 CEPs, SEPs, and DEPs in horse and donkey sperm (Figure 7A). Subsequently, correlation matrices were constructed to assess the relationships between CEPs, SEPs, and DEPs in horse and donkey sperm and their viability. Our findings revealed that the percentage of static and slow sperm in horses was positively correlated with the HBA, ACADSB, and LIPT1 proteins but negatively correlated with the EVI5 and C2H1orf50 proteins (*p* < 0.05). The percentage of progressive sperm was positively correlated with the AKAP4, GSTM3, TUBB4B, ODF1, ODF2, AKAP3, CABYR, CYCT, MSTO1, CIMAPIA, S100A12, CFAP99, and IQCA1L proteins but negatively correlated with the KLK1E2 and GALNTL5 proteins (*p* < 0.05). The percentage of motile sperm was positively correlated with the EVI5 and C2H1orf50 proteins but negatively correlated with the HBA, ACADSB, and LIPT 11 proteins (*p* < 0.05) (Figure 7B). In donkey sperm, the percentages of static and slow sperm were positively correlated with the ICAM3, NCF1, KRT10, EEF2, and IQCA1L proteins but negatively correlated with the ODF1, AKAP3, ZPBP, GALNTL5, and ACADSB proteins (*p* < 0.05). The percentages of progressive and motile sperm were positively correlated with the KLK1E2, EVI5, and C2H1orf50 proteins but negatively correlated with the TIAM2 and DMAC1 proteins (*p* < 0.05) (Figure 7C). This finding suggests a potential correlation between sperm proteins and sperm vitality in horses and donkeys.

## 4. Discussion

The composition and expression of sperm proteins determine male sperm viability and fertility [42]. Horses and donkeys are two prominent livestock species with reproductive characteristics that have long been a subject of great interest [43]. In this study, we conducted a comprehensive quantitative analysis of sperm proteins in stallions and male donkeys. The 4D-DIA proteomics strategy represents the most powerful technology for globally analyzing signal networks within defined biological systems [23,29,44]. In livestock reproduction, proteomics has been utilized to elucidate the molecular underpinnings of sperm freezability, sperm motility, and fertility [45,46,47]. Nevertheless, there has yet to be an in-depth exploration of the sperm proteome in horses and donkeys. Herein, we compared alterations in the composition and expression of sperm proteins between horses and donkeys while exploring their correlation with sperm vitality, aiming to reveal differences in reproductive physiology between these two species.

Regarding sperm viability, we observed that the percentage of progressive and motile sperm in donkeys surpassed that in horses, while the percentage of static and slow sperm was lower than that in horse sperm. This finding is in line with the results of Sabrina et al., who identified significant differences in kinematic parameters between donkeys’ sperm and horses’ sperm. All kinematic parameters were notably greater in donkey sperm than in horse sperm, with donkey sperm exhibiting greater speed and straighter trajectories than horse sperm [48]. Miro et al. also reported that donkey sperm exhibited a greater velocity than horse sperm [49]. This disparity may be intricately linked to variations in the composition and expression of sperm proteins.

In this study, 4D-DIA was employed to generate the most recent protein profiles for horse and donkey sperm, resulting in the identification of 3436 proteins in horse sperm and 3404 proteins in donkey sperm. These numbers exhibited slight differences from those reported by previous researchers for horse and donkey sperm. Swegen et al. identified 1030 proteins in horse sperm, while Jie Yu et al. detected 2682 proteins in donkey sperm [50,51]. Both figures are lower than those revealed in our study. This discrepancy may be attributed to methodological differences, as advanced techniques can uncover subtly expressed proteins within horse and donkey spermatozoa.

We observed a remarkable similarity in the top 10 highly expressed proteins in horse and donkey sperm, which were AKAP4, CRISP3, GSTM3, TUBB4B, KLKIE2, ODF1, GAPDHS, ODF2, AKAP3, and ZPBP. This suggests a potential similarity in the primary protein constituents of horse and donkey sperm. These proteins have been implicated in critical functions within sperm physiology. For instance, Han et al. demonstrated that the AKAP4 protein is predominantly localized to the fibrous sheath of sperm flagella; its absence results in a reduced sperm count and diminished motility [52]. Additionally, de Almeida reported that the AKAP4 protein plays an essential role in sperm motility and identified its precursor proAKAP4 as a putative biomarker for assessing sperm quality [53]. Marta Dordas proposed that the concentration of AKAP4 is positively correlated with progressive sperm motion [54]. This observation may account for our experimental findings, wherein the expression of AKAP4 protein was greater in donkey sperm than in horse sperm, resulting in a greater degree of progressive movement in donkey sperm. Moreover, the high expression of AKAP4 protein in horse and donkey sperm can also function as a biomarker for the sperm of these species [15]. In this investigation, through a comprehensive literature review, we ascertained the precise localization of known CEPs and SEPs within horse and donkey sperm. We have identified that the majority of proteins are localized in the tail or mitochondria of sperm. For instance, GSTM3 is located in the sperm tail, AKAP3 is located in the principal portion of sperm, ODF1 is located within the midsection of sperm, GAPDHS is located in the sperm fiber sheath, and ODF2 is located in the mitochondrial sheath at the tail of sperm. CRISP3 is localized in the postacrosome region of the sperm head and translocates to the anterior end of the tail following acrosome reaction. Immunohistochemistry has confirmed the localization of ZPBP within the acrosome of sperm et al. [18,55,56,57,58,59,60]. These proteins are predominantly localized in the sperm flagellar sheath or mitochondria, with the former directly implicated in sperm motility [15,16,61]. Meanwhile, the sperm mitochondria may generate ATP via the tricarboxylic acid (TCA) cycle and oxidative phosphorylation (OXPHOS), which is indispensable for sperm motility [16,62,63]. Furthermore, in addition to CRISP3 and KLK1E2, eight other proteins were expressed at lower levels in horse sperm than in donkey sperm. The diminished expression of these proteins may suggest a potential weakening of either the tail or mitochondrial sheath in horse sperm cells, which could account for their relatively lower observed viability parameters when contrasted with those of donkey sperm.

In addition to the aforementioned CEPs, we also identified SEPs in horse and donkey sperm. Specifically, 73 proteins were expressed in horse sperm, with the top 10 being CABYR, CIMIP2A, HBA, CYCT, MSTO1, CIMAP1A, SPMIP9, MMP23B, ANKFN1, and GPX5. The protein CABYR is primarily localized in the principal piece of the sperm flagellar fiber sheath and is closely associated with the formation of the sperm flagellar fiber sheath and sperm motility [61]. Research by Young et al. revealed that the deletion of the CABYR gene leads to alterations in sperm flagellar morphology and reduced vitality in male mice [16]. This finding indicates a potential close relationship between horse sperm function and this protein. Conversely, donkey sperm do not rely on this protein, suggesting that there may be other proteins that play similar roles. In total, 43 distinct proteins were identified in donkey sperm, with the top 10 most highly expressed proteins being ICAM3, TIAM2, C16orf89, NCF1, DHRS2, LEXM, SKAP2, DMAC1, TLCD3B, and KRT10. TIAM2 is particularly linked to sperm movement, while mutations in this protein can impair sperm motility [64]. This finding suggested that TIAM2 plays an important role in donkey sperm but not in horse sperm, indicating differences in their protein composition. However, in this study, we exclusively observed the specific expression of CABYR protein in horse sperm, while no distinctive expression of TIAM2 protein was detected in donkey sperm. This discrepancy may be attributed to the potentially low level of TIAM2 protein expression in donkey sperm. Nevertheless, both CABYR and TIAM2 can serve as biomarkers for horse and donkey sperm, respectively, and are also pivotal proteins associated with sperm viability in each species.

The distribution of identified DEPs in GO enrichment exhibited a comparable pattern to that observed in the protein profiles of human and mouse sperm [65,66]. The predominant functions of sperm proteins are associated with protein transport, signal transduction, and GTP binding, indicating that the protein structure of horse and donkey sperm is similar to that of other mammals [67]. Furthermore, KEGG analysis revealed a significantly greater proportion of signaling pathways related to steroid hormone biosynthesis and metabolism of xenobiotics by cytochrome P450 in the DEP of donkey sperm compared to that of horse sperm. This is due to the high expression of DEP protein genes in donkey sperm, which are primarily associated with mitochondrial functions in sperm cells. Mitochondria play a crucial role in various cellular activities, including the biosynthesis of steroid hormones and the metabolism of exogenous substances by cytochrome P450, among others [68,69]. This provides an explanation for the higher motility observed in donkey sperm compared to horse sperm. Given that all horses and donkeys included in this study were bred and raised under uniform conditions, differences in sperm proteins between these species primarily stemmed from genetic background effects. Thus, this identification underscores the substantial impact of disparities in protein abundance on sperm function between species with distinct genetic backgrounds.

In the context of horse sperm CEPS, SEPs, and DEPs and their relationship with sperm vitality, the expression of 13 proteins with diverse functions (AKAP4, GSTM3, TUBB4B, ODF1, ODF2, AKAP3, CABYR, CYCT, MSTO1, CIMAP1A, S100A12, CFAP99, and IQCA1L) exhibited a positive correlation with the percentage of motility and progression in horse sperm. Conversely, the KLK1E2 and GALNTL5 proteins were negatively correlated with these variables. Concerning the association between donkey sperm protein and sperm vitality, the expression of three proteins, KLK1E2, EVI5, and C2H1orf50, was positively correlated with the percentage of motility and progression in donkey sperm, while TIAM2 and DMAC1 were negatively correlated with these parameters. This aligns with the findings of most researchers [15,70,71]. However, Ashwitha’s hypothesis that the upregulation of GSTM3 protein leads to decreased bull sperm viability contradicts our experimental results [55]. It is postulated that such a discrepancy may be related to the differing functionalities of GSTM3 across species.

These findings offer valuable insights for a deeper understanding of the reproductive characteristics of horses and donkeys. However, this study solely conducted an initial analysis of sperm protein composition and motility, necessitating further comprehensive research to explore the specific functions of individual proteins and their roles in sperm motility. Furthermore, future investigations could delve more extensively into discussions regarding the reproductive characteristics of horses and donkeys from genomic and transcriptomic perspectives, among others. In summary, these investigations will contribute to an enhanced understanding of life’s enigmas and propel the continual progression of reproductive biology.

## 5. Conclusions

In conclusion, this study compared the protein expression profiles in horse and donkey sperm and analyzed the correlation between sperm proteins and sperm viability, revealing the potential mechanisms of differences in sperm viability between horses and donkeys at the molecular level, providing valuable reference for the development of genetic breeding in the field of equine animals. In addition, this study on the molecular diversity of sperm proteins in horses and donkeys will also help researchers to gain a deeper understanding of the biological characteristics of sperm between different species.

## Data Availability

All data are accessible through the article and its Supplementary Materials file or upon request from the authors.

## Supplementary Materials

The following supporting information can be downloaded at: https://www.mdpi.com/article/10.3390/ani14152237/s1, Figure S1: Uncropped Western blot figures; Table S1: Gray values of Western blot; Table S2: Reference genome information; Table S3: Proteome map statistics.
