# Supplementary figures and images for: Comparative Study on the Sperm Proteomes of Horses and Donkeys
Source: Animals (Basel). 2024 Jul 31;14(15):2237. doi: 10.3390/ani14152237 (PMC11311092; doi:10.3390/ani14152237)

AKAP4

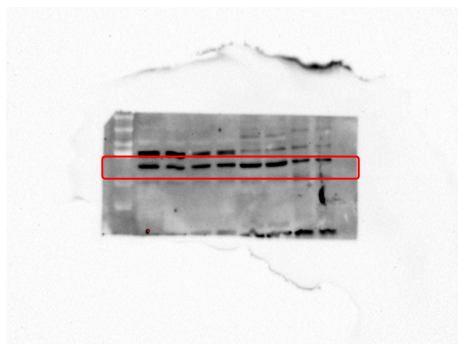

TUBB

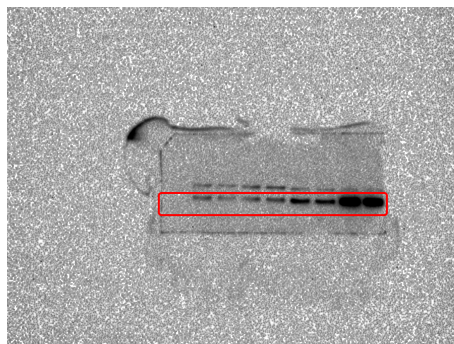

CABYR

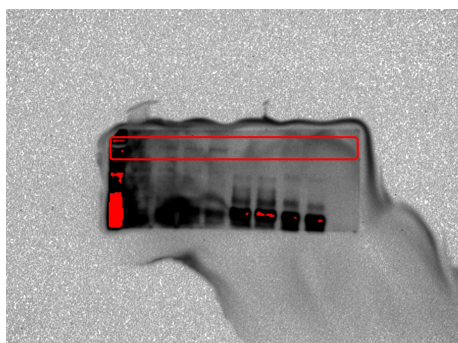

TUBB

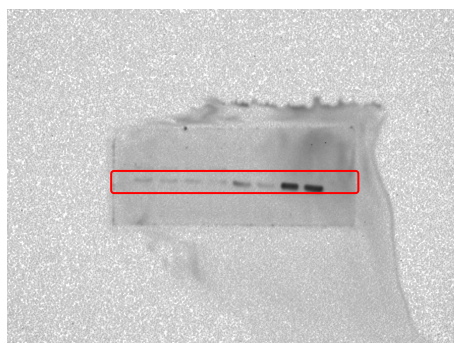

ACADSB

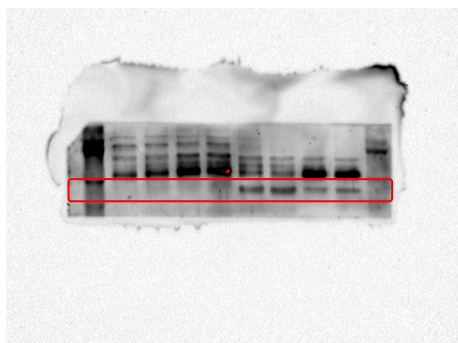

TUBB

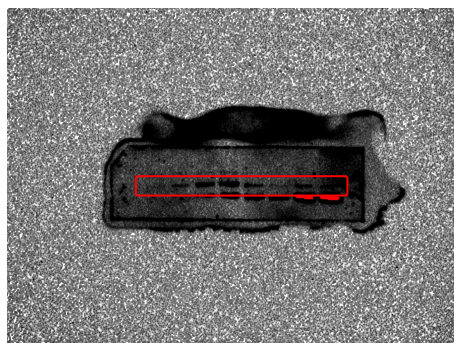

S100A12

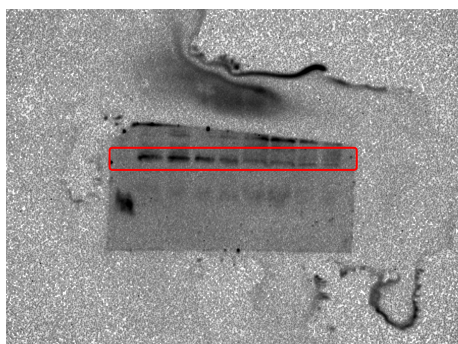

TUBB

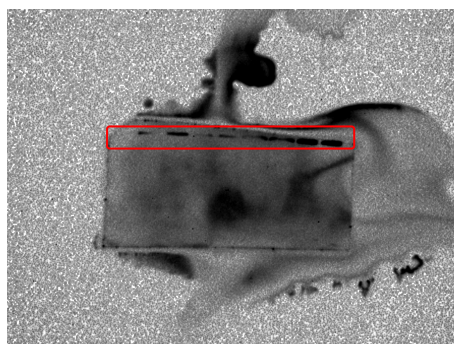

Supplement: Supplementary file 1 [file animals-14-02237-s001.zip › Figure S1 Uncropped western blot figures.pdf]
